# The Effects of Heated Tobacco Products on Oral Health and Quality of Life: An Observational Cross-Sectional Study

**DOI:** 10.3390/healthcare14101297

**Published:** 2026-05-11

**Authors:** Ana Glavina, Anđela Gravić, Josipa Demo, Dinko Martinović, Antonija Tadin, Stjepanka Lešić, Daniela Šupe-Domić

**Affiliations:** 1 Department of Dental Medicine, University Hospital of Split, 21000 Split, Croatia; atadin@mefst.hr; 2 Department of Oral Medicine, Study of Dental Medicine, School of Medicine, University of Split, 21000 Split, Croatia; gravica10@gmail.com (A.G.); josipa.de17@gmail.com (J.D.); 3 Department of Maxillofacial Surgery, University Hospital of Split, 21000 Split, Croatia; 4 Department of Pathophysiology, School of Medicine, University of Split, 21000 Split, Croatia; 5 Department of Restorative Dental Medicine and Endodontics, Study of Dental Medicine, School of Medicine, University of Split, 21000 Split, Croatia; 6 Department of Dental Medicine, Faculty of Dental Medicine and Health Osijek, Josip Juraj Strossmayer University of Osijek, 31000 Osijek, Croatia; 7 Department of Medical Laboratory Diagnostics, University Hospital of Split, 21000 Split, Croatia; daniela.supedomic@gmail.com; 8 Department of Health Studies, University of Split, 21000 Split, Croatia

**Keywords:** smoking, heated tobacco products, saliva, halitosis, DMFT index, oral lesions, quality of life

## Abstract

Background/Objectives: Over the past decade, the popularity of smokeless tobacco products, particularly heated tobacco products (HTPs), has increased among adolescents and young adults. This study aimed to determine the effects of HTPs and conventional cigarettes on oral health and quality of life (QoL). Methods: This stratified cross-sectional study included 90 participants divided into three groups: conventional cigarette smokers (*N* = 30), HTP users (*N* = 30), and non-smokers (*N* = 30). Sociodemographic data and oral-medical status [salivary pH, halitosis, sialometric measurements, Decayed, Missing, Filled Teeth (DMFT) index, and oral lesions] were recorded for all participants. Results: There was no statistically significant difference in salivary pH, unstimulated whole saliva (UWS), or stimulated whole saliva (SWS) among the three groups (*p* = 0.343, *p* = 0.982, and *p* = 0.793, respectively). There was also no statistically significant difference in DMFT index values (*p* = 0.495) or total QoL (*p* = 0.856) among the groups. However, there was a statistically significant difference in halitosis among the groups (*p* < 0.0001), with moderate (40.0%, *N* = 12) and strong (33.3%, *N* = 10) halitosis most frequent among HTP users. There was no statistically significant difference in the incidence of pathological oral lesions among the groups (*p* = 0.112), with 63.3% (*N* = 19) among conventional cigarette smokers. Conclusions: HTP users exhibited a higher frequency of moderate and strong halitosis, while conventional cigarette smokers more frequently presented with smoker’s melanosis and lesions located on the hard palate.

## 1. Introduction

According to the World Health Organization (WHO), nicotine-containing tobacco products are addictive and a significant risk factor for cardiovascular and respiratory diseases. They are hazardous not only to smokers but also to passive smokers and can cause numerous immunocompromising conditions [1]. Over the past two decades, the tobacco industry has undergone a major transformation as awareness of the harmful effects and consequences of smoking has increased. “Healthier and safer” alternative forms of smoking have entered the market, most notably heated tobacco products (HTPs) [2]. HTPs heat tobacco without burning it, reaching lower temperatures (250 to 300 °C) than the combustion of tobacco in conventional cigarettes (600 to 800 °C). However, HTPs produce an aerosol containing nicotine and other harmful chemicals (nitrosamines, carbonyl compounds) that are also present in conventional cigarettes, though in lower concentrations [2,3]. One claimed advantage of HTPs is the ability to switch the product on and off independently, giving users a false sense of control over the amount of inhalation or nicotine consumption [4]. Although HTPs produce an aerosol with a lower concentration of harmful chemicals, they still emit pollutants that have been shown to cause cardiovascular and respiratory diseases [1]. As new tobacco products have only been available for a relatively short time, knowledge about their harmful effects on individual health is limited, and claims about the lower risk of HTPs compared to conventional cigarettes are largely based on studies funded by the tobacco industry [1,5]. Due to aggressive marketing campaigns and the promotion of new tobacco products as less harmful alternatives to conventional smoking, the number of new smokers among non-smokers and former smokers of conventional cigarettes has increased. The number of so-called “combination smokers”, those who use both HTPs and conventional cigarettes, is also rising, raising questions about the synergistic effects of these products. HTPs are very popular among younger people and their numbers are increasing daily, which is a cause for concern among health professionals [6].

Nicotine products adversely affect both oral and general health. Smoking is a major risk factor for the development of oral potentially malignant disorders (OPMDs) and oral cancer. Molecular interactions between harmful components of tobacco smoke and the oral epithelium trigger inflammatory reactions, disrupt cellular homeostasis, and promote carcinogenesis. The oral mucosa responds to pathological stimuli with a series of morphological changes, including hyperkeratinisation, hyperkeratosis, atrophy, and metaplasia of the epithelium. Consequently, various clinical conditions associated with tobacco use may develop, such as smoker’s melanosis, nicotine palatitis and stomatitis, leukoedema, and OPMDs such as oral lichenoid lesions (OLLs), leukoplakia, and erythroplakia, which can progress to oral cancer [5,7,8].

Saliva is a unique, complex, and specific secretion that undergoes structural and functional changes in the salivary glands upon contact with tobacco, resulting in reduced saliva production [5]. Nicotine stimulates taste and certain cholinergic receptors, and affects blood flow in the salivary glands [5,9]. Recent studies have confirmed that smoking also negatively affects saliva quality. Toxic substances from tobacco smoke destroy protective substances, enzymes (lysozyme, peroxidase, lactoferrin, agglutinins), and proteins, causing saliva to lose its protective function [10]. Hyposalivation is a major public health problem as it impairs oral function and health. Dry mouth favours the development of inflammation, fungal infections (oral candidiasis), more rapid caries development, halitosis, and functional changes such as problems with chewing, swallowing, and speech [10]. Smoking (conventional cigarettes or HTC) is the second most common cause of halitosis, after periodontal disease, due to changes in the oral microflora and the growth of pathogenic microbes [5,11].

The objective of the study was to determine the effects of HTPs and conventional cigarettes on oral health [Decayed, Missing and Filled Teeth (DMFT), physiological and pathological oral lesions and their topography according to WHO, sialometric measurements, salivary pH, and halitosis] and quality of life (QoL). The hypothesis of this study is that HTP users have poorer oral health and QoL compared to conventional cigarette smokers and non-smokers.

## 2. Materials and Methods

### 2.1. Study Design, Subjects, Inclusion and Exclusion Criteria

This was a stratified cross-sectional study in which the total number of participants (*N* = 90) was divided into three groups: conventional cigarette smokers (*N* = 30), HTP users (*N* = 30), and non-smokers (*N* = 30). Before participation, the study protocol was explained to each participant, and each voluntarily signed an informed consent form. Participants who did not agree to the content of the informed consent form were excluded from further participation. The study was conducted at the Department of Dental Medicine, University Hospital of Split, Split, Croatia, from February 2025 to June 2025. The Ethics Committee of the University Hospital of Split, Split, Croatia, approved the study protocol on 6 February 2025 (Class: 520-03/25-01/17, Reg. No.: 2181-147/01-06/LJ.Z.-25-02), and the study was conducted in accordance with the Declaration of Helsinki. To ensure quality and transparency, the guidelines of the STROBE statement for cross-sectional studies were followed (Table S1) [12].

The inclusion criteria for HTP users were consumption of at least ≥100 heated tobacco sticks since starting smoking and no use of conventional cigarettes in the past six months. The six-month abstinence from conventional cigarette use was applied to distinguish current HTP users; however, prior smoking history was not an exclusion criterion. The inclusion criteria for conventional smokers were consumption of at least ≥100 cigarettes since starting smoking. The control group consisted of subjects with a negative history of smoking throughout their entire lives. This threshold is consistent with commonly used epidemiological definitions of smoking status in previous studies [5,6].

The exclusion criteria for all three groups were:

1. Use of other forms of tobacco (simultaneous use of conventional cigarettes and HTPs, e-cigarettes, or nicotine pouches);
2. Presence of oral or systemic diseases (arterial hypertension, diabetes mellitus (DM), gastro-oesophageal reflux disease, liver disease, thyroid disease, infectious diseases, neurodegenerative diseases, psychiatric diseases, autoimmune diseases, immunocompromised status, or history of transplantation);
3. Use of medications that reduce salivary flow (antiseptics in the past 30 days, antiseptics with alcohol, antibiotics, alpha receptor antagonists, anticholinergics, antidepressants, amphetamines, antihypertensives, antihistamines, antipsychotics, benzodiazepines, hypnotics, opioids, antiparkinsonians, antiepileptics, bronchodilators, H2 receptor antagonists, proton pump inhibitors (PPI), protease inhibitors, retinoids, muscle relaxants, diuretics, sympathomimetics, antispasmodics, hypoglycaemics, statins, analgesics, anti-inflammatory drugs, radiotherapy to the head and neck area, chemotherapy, or biological therapy);
4. Subjects under <18 years of age.

**Figure 1:**
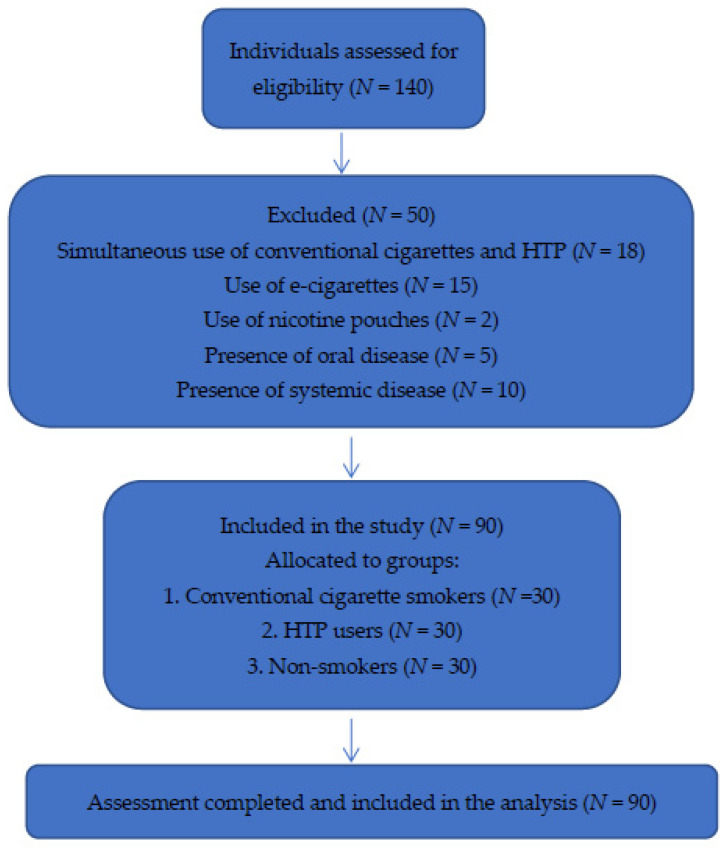
Participant flow diagram. Abbreviation: HTP, heated tobacco product.

The participant selection process is presented in Figure 1.

### 2.2. Bias

Potential sources of bias were considered during the study design and analysis. Selection bias may have occurred due to the use of convenience sampling and recruitment from a single academic institution. Information bias is possible due to self-reported smoking history. To minimise measurement bias, standardised clinical examination protocols and calibrated examiners were used. However, residual confounding may be present, as oral hygiene habits and other lifestyle factors were not fully controlled.

### 2.3. Study Protocol

Sociodemographic data (age, gender, level of education), medical and dental history, DMFT index, and oral-medical status (including changes in the oral mucosa, salivary pH, halitosis, sialometric measurements, and topography of oral lesions according to the WHO scheme) were recorded for all subjects [13]. An intraoral clinical examination was performed by an oral medicine specialist with more than five years of specialist experience (A.G.). Clinical data were collected with the patient seated in the dental unit, illuminated by a professional dental light, and using a standardised dental probe (Devemed GmbH, Tuttlingen, Germany) [13,14].

#### 2.3.1. Sialometric Measurements and Salivary pH

Saliva collection was performed in the morning, between 09:00 and 12:00, to minimise daily fluctuations in salivary secretion. Unstimulated whole saliva (UWS) and stimulated whole saliva (SWS) were collected by spitting into a graduated tube over a five-minute period. Subjects were instructed not to eat, drink, smoke, or perform oral hygiene for at least 90 min before the procedure. During the procedure, subjects were asked not to speak or swallow. A 1.0% solution of vitamin C (1 g ascorbic acid in 1 dcl of water) was used to stimulate salivation. Saliva flow was calculated by dividing the volume of collected saliva by the collection time, and hyposalivation was diagnosed if saliva flow was less than 0.2 mL per minute [15]. Salivary pH was determined using universal indicator paper, which was placed on the dorsum of the tongue with sterile dental forceps in direct contact with the saliva. Subjects were instructed to keep their mouths closed for one minute. The colour of the saliva-soaked indicator paper was then compared to the provided colour scale or pH table. pH values below 7.0 are considered acidic, while values above 7.0 are considered alkaline [16].

#### 2.3.2. Halitosis

Halitosis was assessed using the organoleptic method. Subjects were instructed not to eat, drink, smoke, or perform oral hygiene for at least 90 min before the test. During the procedure, subjects kept their mouths closed for two minutes and breathed through their noses. After two minutes, they exhaled slowly through their mouths, 5–10 cm from the examiner’s nose, and the examiner assessed the odour on a scale from 0 to 5: 0—no noticeable odour; 1—barely noticeable odour; 2—mild but noticeable odour; 3—moderate odour; 4—strong odour; 5—pronounced odour, according to Rosenberg et al. [17].

#### 2.3.3. DMFT Index

The DMFT index is used to assess and monitor oral health in a population. It is the standard and most important index in epidemiological studies for evaluating population health status. The DMFT index records the number of teeth affected by caries (code D), the number of teeth extracted due to caries (code M), and the number of teeth with fillings (code F). It is calculated by summing all teeth with the codes D, M, and F (D + M + F), with each code representing the number of teeth in a specific category. The main disadvantage of the DMFT index is its inability to record carious lesions on teeth that are missing or have fillings, as well as its inability to record reasons for tooth loss other than caries. During clinical examination and evaluation, all teeth except third molars were included, and the visual–tactile method was used with a standard dental probe (Devemed GmbH, Tuttlingen, Germany) under conventional dental lighting [13,14].

#### 2.3.4. Oral Lesions According to the WHO Topographic Scheme

A visual method was used to detect pathological and physiological changes in the oral mucous membrane, which were recorded using the WHO topographic scheme [18].

### 2.4. Instruments

#### Croatian Version of the Oral Health Impact Profile (OHIP-CRO14)

The OHIP-CRO14 was used to assess the impact of oral health on overall subject satisfaction. The questionnaire comprised 14 questions divided into seven groups: functional limitation, physical pain, psychological disability, physical disability, psychological discomfort, social disability, and handicap. Subjects answered the questions using a Likert scale from 0 to 4, where 0 represents the absence of a problem and 4 the greatest possible problem. The total score was calculated by summing the response values, with higher scores indicating poorer patient satisfaction and lower scores indicating greater patient satisfaction [19].

### 2.5. Power Analysis

The sample size was determined using a power analysis for a one-way analysis of variance (ANOVA) with three independent groups. Cohen’s f = 0.40 was used as the expected effect size, representing a large effect. The probability of a type I error was set at 0.05, and the desired power at 90%, ensuring a high probability of detecting a true effect. Based on these parameters, a total of 84 participants were required to achieve the specified power, with 28 participants in each group. This sample size allows reliable detection of differences between groups with an acceptable level of statistical error.

### 2.6. Statistical Analysis

Data for this study were analysed using MedCalc for Windows^®^ (MedCalc Software, Ostend, Belgium, version 23.2.0). Quantitative data are presented as mean ± standard deviation (SD) or median (interquartile range, IQR), depending on the normality of the variable distribution. Qualitative data are expressed as absolute number (N) and percentage (%). Normality of distribution was assessed using the Kolmogorov–Smirnov test. The parametric ANOVA was used to compare quantitative variables with normal distribution, while the non-parametric Kruskal–Wallis test was used to analyse qualitative variables with non-normal distribution. The chi-square (χ^2^) test was used for comparisons between categorical variables. The level of statistical significance was set at *p* < 0.05.

## 3. Results

### 3.1. Sociodemographic Characteristics and Habits

A total of 90 subjects participated in the cross-sectional study, divided into three groups: conventional cigarette smokers (*N* = 30), HTP users (*N* = 30), and non-smokers (*N* = 30). Across the three groups, 73.3% (*N* = 66) were women, with an age range of 22–40 years (median = 25). There was no statistically significant difference in gender or age between the groups (*p* = 0.999, *p* = 0.054).

The majority of subjects, 80.0% (*N* = 72), were highly educated, mainly colleagues from the School of Medicine, University of Split, Split, Croatia (*p* = 0.232). There was a statistically significant difference in the duration of smoking experience between HTP users and conventional cigarette smokers (*p* = 0.044). Specifically, 43.3% (*N* = 13) of HTP users had used heated tobacco sticks for 1 to 2 years, while 33.3% (*N* = 10) of conventional cigarette smokers had smoked for 2 to 5 years. Among HTP users, 30% (*N* = 9) consumed more than 15 heated tobacco sticks per day, whereas 30% (*N* = 9) of conventional cigarette smokers consumed fewer than 5 cigarettes per day (*p* = 0.612) (Table 1).

### 3.2. Salivary pH and Sialometric Measurements

**Figure 2:**
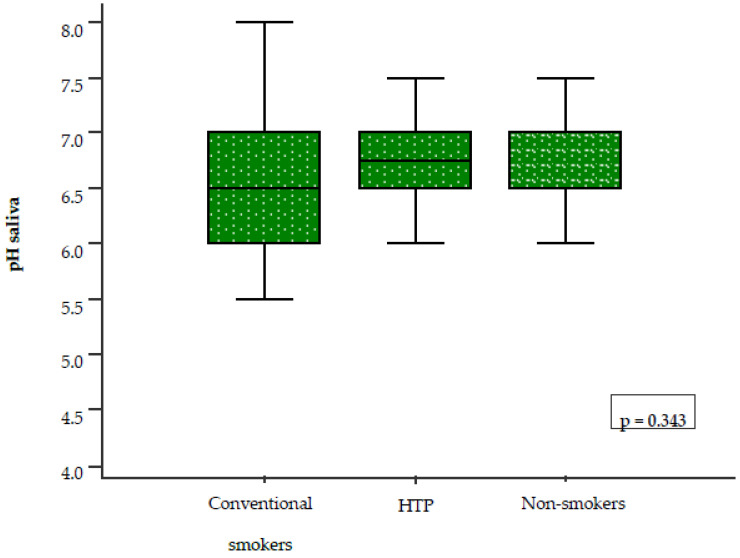
Comparison of salivary pH among the three groups. The Kruskal–Wallis test was used for group comparison. Abbreviation: HTP, heated tobacco product.

No statistically significant difference in salivary pH was found among the three groups (*p* = 0.343). However, lower median pH values, indicating more acidic saliva, were observed in conventional cigarette smokers (pH = 6.5) compared to HTP users (pH = 6.75) and non-smokers (pH = 6.75) (Figure 2).

**Figure 3:**
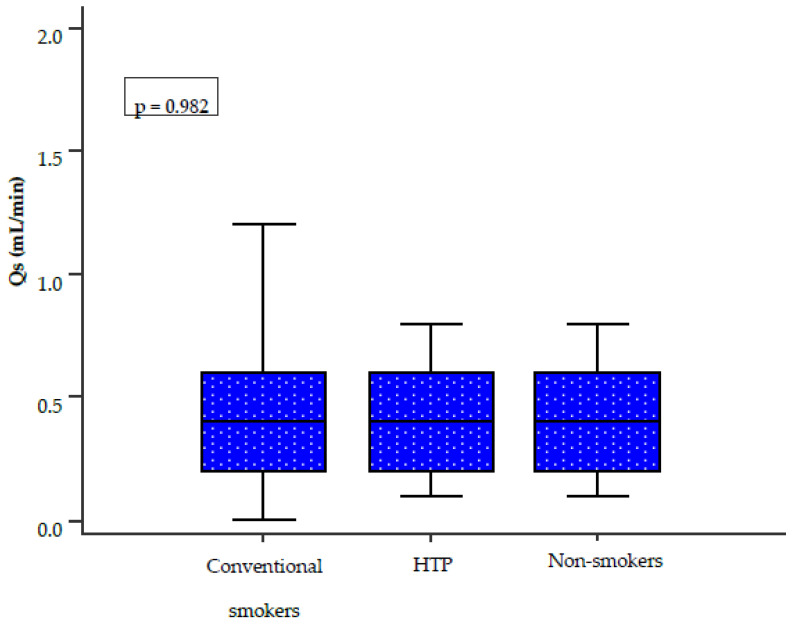
Comparison of UWS among the three groups. The Kruskal–Wallis test was used for group comparisons. Abbreviations: UWS (Qs), unstimulated whole saliva; HTP, heated tobacco product.

The measured UWS showed no statistically significant differences among the three groups (0.4 mL/min vs. 0.4 mL/min vs. 0.4 mL/min; *p* = 0.982) (Figure 3).

**Figure 4:**
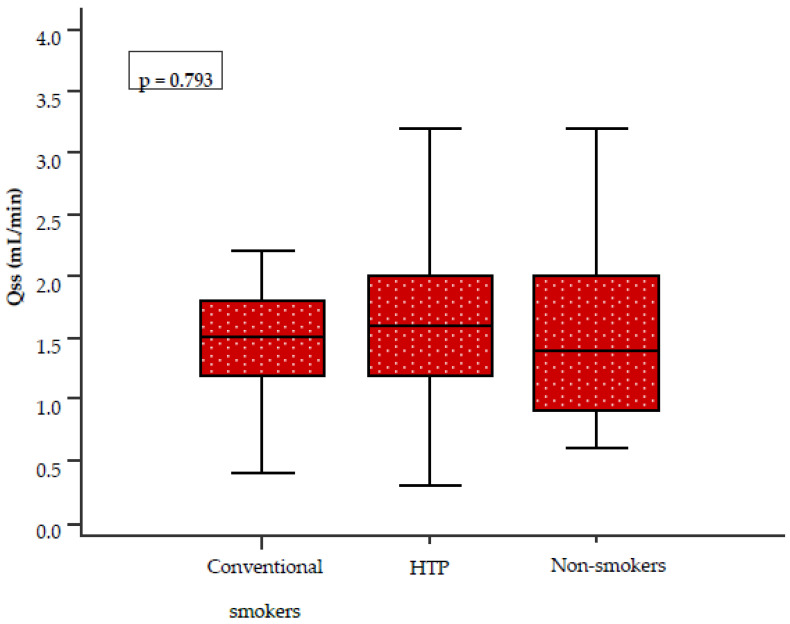
Comparison of SWS among the three groups. The Kruskal–Wallis test was used for group comparisons. Abbreviations: SWS (Qss), stimulated whole saliva; HTP, heated tobacco product.

There was no statistically significant difference in SWS among the three groups (*p* = 0.793). However, the measured values were lower in conventional cigarette smokers than in HTP users (1.51 mL/min vs. 1.6 mL/min; see Figure 4).

### 3.3. DMFT Index, QoL and Halitosis

There was no statistically significant difference in DMFT index values (*p* = 0.495) or total QoL, as assessed by the OHIP-CRO14 questionnaire (*p* = 0.856), among the three groups. However, conventional cigarette smokers had the highest total OHIP-CRO14 scores, indicating the poorest QoL compared to non-smokers and HTP users. There were no statistically significant differences in the individual categories of the OHIP-CRO14 questionnaire (Table S2).

There was a statistically significant difference in the frequency of halitosis among the three groups (*p* < 0.0001). Moderate halitosis, as assessed by the organoleptic method, was observed in an equal percentage of HTP users and conventional cigarette smokers (40.0%, *N* = 12), while only barely noticeable halitosis was recorded in 46.7% (*N* = 14) of non-smokers. In addition to moderate halitosis, 33.3% (*N* = 10) of HTP users exhibited strong halitosis (Table 2).

### 3.4. Changes in the Oral Mucosa According to the WHO Topographic Scheme

#### 3.4.1. Oral Lesions

There was no statistically significant difference in the incidence of pathological oral lesions among the three groups (*p* = 0.112). However, the number of pathological oral lesions was higher among conventional cigarette smokers (63.3%, *N* = 19) than among HTP users (40.0%, *N* = 12). There was a statistically significant difference in the incidence of smoker’s melanosis between conventional cigarette smokers and HTP users (*p* = 0.008). *Lingua villosa alba* was detected in 33.3% (*N* = 10) of conventional cigarette smokers, while HTP users and non-smokers showed a lower incidence (26.7%, *N* = 8; *p* = 0.805). Pathological changes in the form of morsicatio were detected in more HTP users (16.7%, *N* = 5) than in conventional cigarette smokers (6.7%, *N* = 2; *p* = 0.329). Leukoedema was observed in half of the HTP users (50.0%, *N* = 15) compared to 36.7% (*N* = 11) of conventional cigarette smokers (*p* = 0.581) (Table 3).

#### 3.4.2. Locations of Oral Lesions

**Figure 5:**
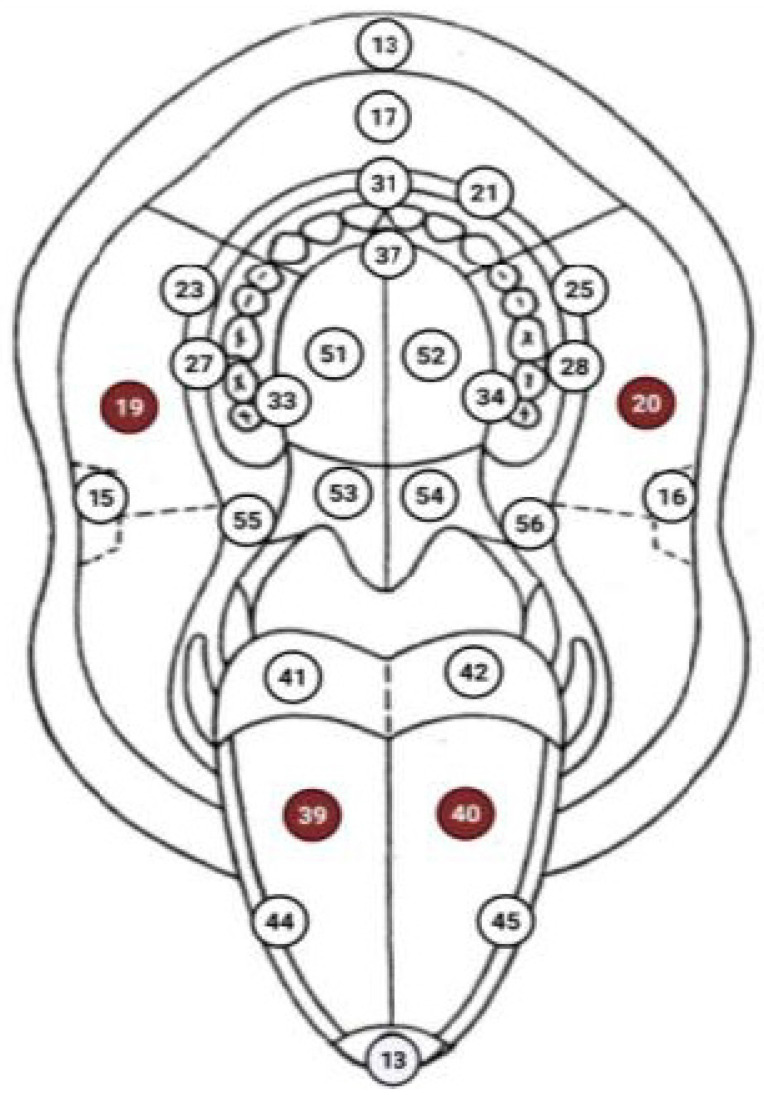
Locations according to WHO topography. The numbers (19, 20, 39, and 40) indicate anatomical locations in the oral cavity according to the WHO topographic scheme. Red circles show the distribution and frequency of oral lesions in the examined groups. Abbreviation: WHO, World Health Organization.

The highest number of total oral lesions (pathological and physiological) in all three groups was found on the right buccal mucosa (location 19 according to the WHO topographic scheme) and the left buccal mucosa (location 20 according to the WHO topographic scheme), with a prevalence of 53.3% (*N* = 48). Although the difference among the three groups was not statistically significant (*p* = 0.391), oral lesions at these locations were most common in HTP users (63.3%, *N* = 19). Oral lesions on the dorsum of the tongue (locations 39 and 40 according to the WHO topographic scheme) also did not show a statistically significant difference among the three groups (*p* = 0.550), although a higher prevalence was recorded among conventional cigarette smokers (46.7%, *N* = 14) compared to HTP users (33.3%, *N* = 10) and non-smokers (43.3%, *N* = 13). There was a statistically significant difference in the occurrence of oral lesions on the hard palate (locations 51 and 52 according to the WHO topographic scheme) (*p* = 0.044), with lesions at these sites recorded exclusively in conventional cigarette smokers (10.0%, *N* = 3), while no cases were found in HTP users or non-smokers (Figure 5).

## 4. Discussion

The study results showed that most subjects were women (73.3%, *N* = 66). This does not necessarily reflect the actual gender distribution of HTP users in Croatia but is more likely due to the sampling method and participant recruitment. Specifically, a convenience sampling approach was used, and most participants were recruited from the School of Medicine, University of Split, Split, Croatia, which may have contributed to a higher response rate among women. Globally, the prevalence of conventional cigarette and HTP use is higher among men. A large cross-sectional study of 7714 subjects in Japan reported HTP use in 5.0% of men and 2.2% of women, suggesting higher uptake among men and young adults in their twenties and thirties [20]. Our findings are partially consistent with this, as most HTP users in our study were young adults in their twenties (median age = 23). However, the predominance of female participants is a limitation and reduces the generalisability of the findings. Similar gender distributions were reported in studies by Božac E et al. [21] and Sever E et al. [5], suggesting that this imbalance may be related to higher participation rates and greater health awareness among women rather than true population characteristics [22]. Additionally, most subjects in all three groups (80.0%, *N* = 72) were highly educated, predominantly affiliated with the School of Medicine, University of Split, Split, Croatia. This further limits representativeness, as such populations may demonstrate higher oral health awareness and different health-related behaviours compared to the general population.

There was a statistically significant difference in smoking duration between HTP users and conventional cigarette smokers (*p* = 0.044). Specifically, 43.3% (*N* = 13) of HTP users had used these products for 1 to 2 years, while 33.3% (*N* = 10) of conventional cigarette smokers had a smoking history of 2 to 5 years. This likely reflects the relatively recent introduction of HTPs to the market, which is a limitation of this study. Although no statistically significant difference was observed in the number of cigarettes or heated tobacco sticks consumed daily between the two groups (*p* = 0.612), 30.0% (*N* = 9) of HTP users reported consuming more than 15 heated tobacco sticks per day, while the same proportion of conventional cigarette smokers (30.0%, *N* = 9) reported smoking fewer than 5 cigarettes per day. These results suggest differences in consumption patterns between the two groups, although the clinical relevance of these differences requires further investigation. More frequent use of heated tobacco sticks may be due to the perception of greater control over tobacco consumption.

Currently, only a limited number of studies have investigated salivary pH among HTP users, conventional cigarette smokers, and non-smokers. In our study, no statistically significant differences in salivary pH values were observed between the three groups (*p* = 0.343). However, lower salivary pH values, indicating a more acidic oral environment, were found among conventional cigarette smokers. Our results are partially consistent with those of a large cohort study conducted in Iran, which included 510 participants and reported a statistically significant difference in salivary pH between conventional cigarette smokers and non-smokers (*p* = 0.018) [23]. The results for UWS and SWS did not show statistically significant differences between the three groups (*p* = 0.982, *p* = 0.793). However, lower SWS values were observed in conventional cigarette smokers compared to HTP users. These findings are not fully consistent with those reported by Sever E et al., conducted at the Clinic of Dental Medicine, Clinical Hospital Centre Rijeka, Rijeka, Croatia. In that study, which included 60 participants divided into three equal groups, the influence of HTP and conventional cigarette use on UWS was evaluated [5]. The authors reported hyposalivation in HTP users, although to a lesser extent than in conventional cigarette smokers. In contrast, our study found comparable UWS values across all three groups. The observed differences between the studies may be related to variations in smoking history, study populations, and methodology. In our study, the average smoking duration among HTP users ranged from one to two years, while for conventional cigarette smokers it ranged from two to five years. In contrast, Sever E et al. reported a substantially longer average smoking duration of approximately ten years in both groups. The literature on the long-term effects of smoking on salivary flow remains inconsistent. Khan GJ et al. [24] reported that long-term smoking has no significant effect on salivary flow, whereas Rad M et al. [25] demonstrated significant differences in both salivary flow rates and the subjective sensation of dry mouth between smokers and non-smokers. Similarly, Petrušić N et al. found statistically significant differences in whole saliva between smokers and non-smokers, with hyposalivation increasing proportionally with smoking duration [9].

The DMFT index showed no statistically significant difference among the three groups. This result may be influenced by the relatively short duration of tobacco use (both HTP and conventional) in our sample, as well as the higher proportion of female participants, who generally demonstrate greater engagement in oral health behaviours [22]. However, these factors should be interpreted with caution, as their impact on caries experience cannot be fully established within a cross-sectional design. Our findings are consistent with those of Božac E et al., who also reported no statistically significant differences in the occurrence of new carious lesions between HTP users and conventional cigarette smokers [21]. In contrast, numerous studies have demonstrated a positive association between conventional smoking and dental caries. Axelson P et al., in a multi-age cohort study, reported a statistically significant correlation between the use of conventional tobacco products and the development of new carious lesions [26], and similar findings were reported by Jiang X et al. [27]. From a mechanistic perspective, an in vitro study by Huang R et al. suggested that nicotine may promote co-aggregation of cariogenic bacteria and contribute to the formation of a more mature, thicker biofilm, potentially increasing caries risk [28].

The results of our study did not show a statistically significant difference in total QoL (*p* = 0.856) or in individual OHIP-CRO14 domains among the three groups. However, conventional cigarette smokers consistently exhibited the highest OHIP-CRO14 scores, indicating a trend towards poorer oral health-related quality of life (OHRQoL). Although not statistically significant, this pattern may still be clinically relevant, suggesting that cumulative exposure to tobacco, reflected in a longer smoking history among conventional cigarette smokers, could have a measurable impact on patients’ perceived oral health. From a clinical perspective, these findings highlight that even in the absence of statistically significant differences, dental practitioners should remain attentive to subjective oral health complaints in smokers, as these may precede clinically detectable pathology. The trend towards poorer OHRQoL in conventional smokers may be associated with well-documented effects of tobacco use, including periodontal inflammation, xerostomia, tooth discolouration, and halitosis, all of which can negatively influence daily functioning and psychosocial well-being. Although HTP users did not report worse QoL compared to non-smokers, this does not necessarily indicate the absence of risk. One possible explanation is the shorter duration of HTP use, which may delay the onset of subjective symptoms. Additionally, mechanisms such as reduced salivary flow, alterations in oral microbiota, and the presence of volatile sulphur compounds could contribute to halitosis in HTP users. Even though HTPs are often marketed as less harmful alternatives, their aerosol still contains nicotine and other irritants that may affect oral tissues and microbial balance. Currently, there are no published studies directly comparing OHRQoL using the OHIP-CRO14 among HTP users, conventional cigarette smokers, and non-smokers, which limits direct contextualisation of our findings. Nevertheless, our results are consistent with previous studies demonstrating poorer QoL in conventional cigarette smokers. A cross-sectional study conducted in Nepal with 250 participants reported significantly worse total QoL among conventional cigarette smokers, particularly in domains such as functional limitation, physical pain, psychological disability, and social disability [29]. Similarly, a cross-sectional study from Saudi Arabia including 520 participants found that conventional cigarette smokers had higher OHIP-14 scores across nearly all domains, indicating poorer QoL, with statistically significant differences in most categories except overall life satisfaction [30]. The lack of statistical significance may also be influenced by sample size and variability within groups.

The results of our study showed a statistically significant difference in the occurrence of halitosis among the three groups (*p* < 0.0001). Moderate halitosis was observed in an equal proportion of HTP users and conventional cigarette smokers (40.0%, *N* = 12), while in the control group the majority of participants exhibited only barely noticeable halitosis (46.7%, *N* = 14). Notably, a substantial proportion of HTP users (33.3%, *N* = 10) presented with strong halitosis, highlighting that the use of HTPs is not without noticeable oral health consequences. Clinically, halitosis is an important patient-centred outcome, as it can significantly affect social interactions, self-confidence, and overall QoL. The high prevalence and intensity of halitosis observed in both smoking groups suggest that dental practitioners should actively screen for and address this condition in tobacco users, including those who use HTPs and may perceive them as a safer alternative. Several mechanisms may explain the association between tobacco product use and halitosis. Both conventional cigarettes and HTPs can reduce salivary flow, leading to xerostomia and impaired oral clearance. In addition, tobacco use promotes shifts in the oral microbiota towards anaerobic, proteolytic bacteria that produce volatile sulphur compounds, the primary contributors to oral malodour. Although HTPs do not involve combustion, their aerosol still contains nicotine and other chemical compounds that may alter the oral environment, facilitating similar microbial and biochemical processes. The study by Sever E et al. is partially consistent with our findings, as it also demonstrated statistically significant differences in halitosis between HTP users and conventional cigarette smokers. However, their results indicated a lower frequency and intensity of halitosis among HTP users compared to conventional cigarette smokers [5]. This discrepancy may be attributed to differences in the types of HTP devices used, patterns of consumption, duration of exposure, or variations in participants’ oral hygiene habits and baseline oral health status. These findings suggest that HTP use should not be overlooked as a potential risk factor for halitosis in clinical dental practice.

Tobacco use is a well-established risk factor for a wide range of oral diseases, including potentially malignant and malignant lesions. Inhalation of toxic compounds in tobacco smoke induces inflammatory and structural changes in the oral epithelium, leading to histopathological alterations that may predispose individuals to carcinogenesis [31]. A large cross-sectional study identified smoking as a key contributor not only to malignant and potentially malignant lesions, but also to benign conditions such as nicotine stomatitis (*palatitis nicotinica*) and smoking melanosis [32]. In our study, conventional cigarette smokers showed a higher overall prevalence of pathological oral lesions (63.3%, *N* = 19) compared to HTP users (40.0%, *N* = 12). However, the relatively high frequency among HTP users is noteworthy, particularly given their shorter duration of exposure. This may suggest that even reduced-combustion tobacco products can induce early mucosal changes, which could progress with continued use. A statistically significant difference was observed in the occurrence of smoking melanosis between conventional cigarette smokers and HTP users (*p* = 0.008). This may be explained by differences in thermal degradation and chemical composition between conventional cigarette smoke and HTP aerosol, with combustion products in traditional cigarettes likely exerting a stronger stimulatory effect on melanocytes. Nevertheless, the presence of melanosis in HTP users indicates that these products are not biologically inert and may still trigger pigmentation changes. Benign reactive lesions such as *lingua villosa alba* were more frequent in conventional cigarette smokers (33.3%, *N* = 10) than in HTP users (26.7%, *N* = 8), which may be associated with chronic irritation, altered keratinisation, and changes in oral hygiene habits linked to long-term smoking. In contrast, the higher prevalence of the parafunctional habit morsicatio among HTP users (16.7%, *N* = 5 vs. 6.7%, *N* = 2) may reflect behavioural or psychosocial factors, such as stress, particularly in a younger student population. This highlights the importance of considering not only direct chemical effects but also lifestyle-related contributors to oral mucosal changes. Among physiological variations, leukoedema was the most commonly observed finding, present in 50.0% (*N* = 15) of HTP users and 36.7% (*N* = 11) of conventional cigarette smokers. Although generally considered a benign condition, its higher prevalence in tobacco users (conventional and HTP) may be associated with chronic mucosal irritation and epithelial thickening. Clinically, these findings emphasise the need for thorough oral mucosal examination in all tobacco users, including those who use HTPs. Dental practitioners should be aware that HTP use, despite being perceived as a safer alternative, may still be associated with clinically observable mucosal changes, underscoring the importance of early detection, patient education, and regular follow-up. These results challenge the perception of HTPs as a harmless alternative and support their inclusion in routine risk assessment for oral mucosal pathology.

The highest prevalence of oral lesions was observed on the bilateral buccal mucosa (locations 19 and 20 according to the WHO topographical scheme), accounting for 53.3% (*N* = 48) across all groups. Although intergroup differences were not statistically significant (*p* = 0.391), these lesions were more frequently recorded in HTP users (63.3%, *N* = 19). This distribution may be explained by the buccal mucosa’s increased susceptibility to mechanical irritation, friction, and direct exposure to tobacco aerosols or smoke, making it a common site for reactive and keratotic changes regardless of the type of tobacco product used. The dorsum of the tongue (locations 39 and 40) was the second most commonly affected site, with the highest prevalence observed in conventional cigarette smokers (46.7%, *N* = 14; *p* = 0.550). This may be associated with altered oral microbiota, accumulation of debris, and reduced salivary flow in smokers, all of which contribute to coated tongue and other surface changes. These alterations are also closely linked to halitosis, reinforcing the clinical relevance of tongue examination in tobacco users (conventional and HTP). A statistically significant difference was found in the occurrence of lesions on the hard palate (locations 51 and 52), which were present exclusively in conventional cigarette smokers (10.0%, *N* = 3). This is consistent with the known effects of high-temperature tobacco combustion, as the hard palate is directly exposed to heat and toxic byproducts during smoking. The absence of such lesions in HTP users and non-smokers suggests that thermal injury plays a key role in the development of palatal changes, such as nicotine stomatitis. Clinically, the site-specific distribution of lesions provides useful diagnostic cues for dental practitioners. Lesions on the hard palate may be more indicative of conventional cigarette use, while generalised mucosal changes, particularly on the buccal mucosa, may be observed in both conventional and HTP users. This highlights the importance of a systematic intraoral examination and a detailed patient history, including the type and duration of tobacco use. Our findings partially align with those of Sever E et al., who reported a higher incidence of intraoral changes such as mucosal atrophy, inflammation, erosion, ulceration, morsicatio, and coated tongue in conventional cigarette smokers compared to HTP users. However, as in their study, a direct causal relationship between specific lesions and HTP could not be definitively established. Notably, none of these changes were observed in non-smokers, further supporting the role of tobacco exposure (conventional and HTP) as a contributing factor [5]. Together, these findings suggest that both the type of tobacco product and the mode of exposure (thermal vs. aerosol) influence the locations and nature of oral mucosal changes.

The strength of this study lies in its comprehensive approach, integrating the assessment of salivary pH, sialometric measurements, oral mucosal lesions, and QoL within a single analytical framework. Additionally, the inclusion of HTP users, together with comparisons involving dual-use groups, provides further insight and contributes to a more nuanced understanding of the potential oral health effects associated with different patterns of tobacco use.

A limitation of our study is the relatively small sample size, which restricts the generalisability of the findings to the wider population. Additionally, the sample was not fully representative of the general population, as it consisted predominantly of women and highly educated individuals. This may have introduced selection bias, as these groups are more likely to demonstrate greater awareness of and engagement in oral health care, thereby further influencing the results. The use of a ≥ 100 lifetime consumption threshold may not fully reflect current smoking intensity and could have included occasional users. As smoking-related effects are cumulative, some observed outcomes (e.g., DMFT) in HTP users may partly reflect prior conventional cigarette use rather than the exclusive effects of HTPs. Furthermore, as HTPs are relatively new on the market, most users have shorter exposure histories compared to conventional cigarette smokers. Importantly, potential confounding factors were not fully controlled. Oral hygiene practices (e.g., frequency of tooth brushing, use of dental floss or mouthwash, and regular dental visits), as well as other lifestyle factors such as dietary habits, were not systematically assessed or compared between groups. These variables may have influenced clinical outcomes, including DMFT and other oral health parameters and should therefore be considered when interpreting the results. Further research with larger and more diverse samples, as well as longer and more precisely characterised smoking histories, is needed to better evaluate both the short-term and long-term effects of HTPs on oral health.

## 5. Conclusions

Within the limitations of this cross-sectional study, the findings suggest that the use of HTPs is associated with a similar profile for most assessed oral health parameters (salivary pH, UWS, SWS, DMFT index, and total QoL according to the OHIP-CRO14 questionnaire) compared with conventional cigarette smokers and non-smokers. However, HTP users exhibited a higher frequency of moderate or strong halitosis, which may be clinically relevant in everyday practice. Conventional cigarette smoking remains associated with specific oral changes, particularly smoking melanosis and a higher prevalence of lesions on the hard palate, reinforcing its well-established impact on oral mucosal health. Clinically, these findings highlight the importance of including all forms of tobacco use, including HTPs and dual-use patterns, in routine oral health assessment and patient counselling. Further longitudinal studies with larger and more diverse populations, as well as improved control of confounding factors, are needed to clarify the long-term effects of HTP use on oral health and to distinguish its effects from those of prior conventional cigarette exposure.

## Figures and Tables

**Table 1:** Sociodemographic data and habits of the sample.

Parameter	Total Sample (*N* = 90)	Non-Smokers (*N* = 30)	Conventional Cigarette Smokers (*N* = 30)	HTP Users (*N* = 30)	*p*
Male (*N*, %)	24 (26.7)	8 (26.7)	8 (26.7)	8 (26.7)	0.999
Age (years)	25 (22–40)	25 (24–45)	26 (24–40)	23 (21–26)	0.054
Education level (*N*, %)					
Elementary school	-	-	-	-	-
High school	18 (20.0)	5 (16.7)	9 (30.0)	4 (13.3)	0.232
University	72 (80.0)	25 (83.3)	21 (70.0)	26 (86.7)	
Duration of smoking (*N*, %)					
<1 year	3 (5.0)	-	1 (3.3)	2 (6.7)	
1–2 years	16 (26.7)	-	3 (10.0)	13 (43.3)	
2–5 years	17 (28.3)	-	10 (33.3)	7 (23.3)	
5–10 years	12 (20.0)	-	8 (26.7)	4 (13.3)	0.044
>10 years	12 (20.0)	-	8 (26.7)	4 (13.3)	
Cigarettes/sticks (*N*, %)					
<5 per day	15 (25.0)	-	9 (30.0)	6 (20.0)	
5–10 per day	15 (25.0)	-	8 (26.7)	7 (23.3)	
11–15 per day	16 (26.7)	-	8 (26.7)	8 (26.7)	0.612
>15 per day	14 (23.3)	-	5 (16.7)	9 (30.0)	

All data are presented as whole numbers (percentages) or as medians (interquartile ranges). The chi-square test or Kruskal–Wallis test was used to compare groups. Pairwise group comparisons (Group 1 vs. Group 3; Group 2 vs. Group 3) are shown for descriptive purposes. Abbreviations: N, number; HTP, heated tobacco product.

**Table 2:** Clinical data of the sample.

Parameter	Total Sample (*N* = 90)	Non-Smokers (*N* = 30)	Conventional Cigarette Smokers (*N* = 30)	HTP Users (*N* = 30)	*p*
DMFT index	8 (5–12)	9 (5–13)	8 (6–12)	7.5 (5–10)	0.495
Total OHIP-CRO14	4 (0–9)	4 (2–7)	5 (1–10)	4.5 (0–9)	0.856
Halitosis (*N*, %)					<0.0001
No halitosis	4 (4.4)	1 (3.3)	1 (3.3)	2 (6.7)	
Barely noticeable	19 (21.1)	14 (46.7)	3 (10.0)	2 (6.7)	
Noticeable	20 (22.2)	13 (43.3)	4 (13.3)	3 (10.0)	
Moderate	26 (28.9)	2 (6.7)	12 (40.0)	12 (40.0)	
Strong	18 (20.0)	0	8 (26.7)	10 (33.3)	
Extreme	3 (3.3)	0	2 (6.7)	1 (3.3)	

All data are presented as whole numbers (percentages), arithmetic mean ± standard deviation, or median (interquartile range). The chi-square test or Kruskal–Wallis test was used to compare groups. Abbreviations: N, number; HTP, heated tobacco product; DMFT index, decayed, missing, filled teeth; OHIP-CRO14, Croatian version of the Oral Health Impact Profile-14 questionnaire.

**Table 3:** Physiological and pathological oral lesions.

Parameter	Total Sample (*N* = 90)	Non-Smokers (*N* = 30)	Conventional Cigarette Smokers (*N* = 30)	HTP Users (*N* = 30)	*p*
Physiological lesions	49 (54.4)	17 (56.7)	15 (50.0)	17 (56.7)	0.835
Pathological lesions	43 (47.8)	12 (40.0)	19 (63.3)	12 (40.0)	0.112
St. Fordyce	18 (20.0)	7 (23.3)	6 (20.0)	5 (16.7)	0.811
Leukoedema	39 (43.3)	13 (43.3)	11 (36.7)	15 (50.0)	0.581
*Lingua fissurata*	6 (6.7)	2 (6.7)	2 (6.7)	2 (6.7)	0.999
Keratosis	5 (5.6)	2 (6.7)	2 (6.7)	1 (3.3)	0.809
*Lingua villosa alba*	26 (28.9)	8 (26.7)	10 (33.3)	8 (26.7)	0.805
Morsication	9 (10.0)	2 (6.7)	2 (6.7)	5 (16.7)	0.329
Smoker’s melanosis	7 (7.8)	1 (3.3)	6 (20.0)	0	0.008
*Palatitis nicotinica*	2 (2.2)	0	2 (6.7)	0	0.129
*Glossitis rhombica mediana*	2 (2.2)	1 (3.3)	1 (3.3)	0	0.599
*Cheilitis simplex*	1 (1.1)	0	1 (3.3)	0	0.363
*Palatitis prothetica*	1 (1.1)	0	1 (3.3)	0	0.363
*Lingua geographica*	7 (7.8)	2 (6.7)	3 (10.0)	2 (6.7)	0.856
Ankyloglossia	2 (2.2)	1 (3.3)	1 (3.3)	0	0.599
Exfoliation	1 (1.1)	0	1 (3.3)	0	0.363

All data are presented as whole numbers (percentages). The chi-square test was used to compare groups.

## Data Availability

The data presented in this study are available on request from the corresponding author due to privacy reasons.

## Supplementary Materials

The following supporting information can be downloaded at: https://www.mdpi.com/article/10.3390/healthcare14101297/s1, Table S1: STROBE Statement—Checklist of items that should be included in reports of cross-sectional studies; Table S2: Distribution of responses to the OHIP-CRO14 questionnaire items among non-smokers, conventional cigarette smokers, and HTP users.

## Abbreviations

The following abbreviations are used in this manuscript:

HTP	Heated tobacco product
QoL	Quality of life
DMFT	Decayed, Missing, Filled Teeth
UWS	Unstimulated whole saliva
SWS	Stimulated whole saliva
WHO	World Health Organization
OPMD	Oral potentially malignant disorder
OLL	Oral lichenoid lesion
DM	Diabetes mellitus
PPI	Proton pump inhibitors
OHIP-CRO14	Croatian version of the Oral Health Impact Profile
ANOVA	Analysis of variance
SD	Standard deviation
IQR	Interquartile range
N	Number
OHRQoL	Oral Health-Related Quality of Life
