# Supplementary material for: The Effects of Heated Tobacco Products on Oral Health and Quality of Life: An Observational Cross-Sectional Study
Source: Healthcare (Basel). 2026 May 11;14(10):1297. doi: 10.3390/healthcare14101297 (PMC13205621; doi:10.3390/healthcare14101297)
Supplement: Supplementary file 1 [file healthcare-14-01297-s001.zip › healthcare-4248436-supplementary.pdf]

Table S1. STROBE Statement—Checklist of items that should be included in reports of *cross-sectional studies*.

	Item No	Recommendation	Page No
**Title and abstract**	1	(*a*) Indicate the study's design with a commonly used term in the title or the abstract	**1**
		(*b*) Provide in the abstract an informative and balanced summary of what was done and what was found	**1**
**Introduction**			
Background/rationale	2	Explain the scientific background and rationale for the investigation being reported	**1, 2**
Objectives	3	State specific objectives, including any prespecified hypotheses	**2**
**Methods**			
Study design	4	Present key elements of study design early in the paper	**3**
Setting	5	Describe the setting, locations, and relevant dates, including periods of recruitment, exposure, follow-up, and data collection	**3, 4**
Participants	6	(*a*) Give the eligibility criteria, and the sources and methods of selection of participants	**3, 4**
Variables	7	Clearly define all outcomes, exposures, predictors, potential confounders, and effect modifiers. Give diagnostic criteria, if applicable	**3, 4**
Data sources/ measurement	8*	For each variable of interest, give sources of data and details of methods of assessment (measurement). Describe comparability of assessment methods if there is more than one group	**5, 6**
Bias	9	Describe any efforts to address potential sources of bias	**4**
Study size	10	Explain how the study size was arrived at	**6**
Quantitative variables	11	Explain how quantitative variables were handled in the analyses. If applicable, describe which groupings were chosen and why	**6**
Statistical methods	12	(*a*) Describe all statistical methods, including those used to control for confounding	**6**
		(*b*) Describe any methods used to examine subgroups and interactions	**6**
		(*c*) Explain how missing data were addressed	**NP**
		(*d*) If applicable, describe analytical methods taking account of sampling strategy	**6**
		(*e*) Describe any sensitivity analyses	**NP**
**Results**			
Participants	13*	(a) Report numbers of individuals at each stage of study—eg numbers potentially eligible, examined for eligibility, confirmed eligible, included in the study, completing follow-up, and analysed	**3, 6, 7**
		(b) Give reasons for non-participation at each stage	**4**
		(c) Consider use of a flow diagram	**4**
Descriptive data	14*	(a) Give characteristics of study participants (eg demographic, clinical, social) and information on exposures and potential confounders	**6, 7**
		(b) Indicate number of participants with missing data for each variable of interest	**NP**
Outcome data	15*	Report numbers of outcome events or summary measures	**7-10**
Main results	16	(*a*) Give unadjusted estimates and, if applicable, confounder-adjusted	**7-10**

		estimates and their precision (eg, 95% confidence interval). Make clear which confounders were adjusted for and why they were included	
		(*b*) Report category boundaries when continuous variables were categorized	**7-10**
		(*c*) If relevant, consider translating estimates of relative risk into absolute risk for a meaningful time period	**NP**
Other analyses	17	Report other analyses done—eg analyses of subgroups and interactions, and sensitivity analyses	**NP**
**Discussion**			
Key results	18	Summarise key results with reference to study objectives	**10-18**
Limitations	19	Discuss limitations of the study, taking into account sources of potential bias or imprecision. Discuss both direction and magnitude of any potential bias	**14, 15**
Interpretation	20	Give a cautious overall interpretation of results considering objectives, limitations, multiplicity of analyses, results from similar studies, and other relevant evidence	**10-15**
Generalisability	21	Discuss the generalisability (external validity) of the study results	**10-15**
**Other information**			
Funding	22	Give the source of funding and the role of the funders for the present study and, if applicable, for the original study on which the present article is based	**NP**

*Give information separately for exposed and unexposed groups.

Note: An Explanation and Elaboration article discusses each checklist item and gives methodological background and published examples of transparent reporting. The STROBE checklist is best used in conjunction with this article (freely available on the Web sites of PLoS Medicine at http://www.plosmedicine.org/, Annals of Internal Medicine at http://www.annals.org/, and Epidemiology at http://www.epidem.com/). Information on the STROBE Initiative is available at www.strobe-statement.org.

Table S2. Distribution of responses to the OHIP-CRO14 questionnaire items among non-smokers, conventional cigarette smokers, and HTP users.

**Parameter**	**Total Sample (*N* = 90)**	**Non-smokers (*N* = 30)**	**Conventional Cigarette Smokers (*N* = 30)**	**HTP Users (*N* =30)**	***p****
**(1) Have you had, or do you have, difficulty pronouncing words due to problems with your teeth, mouth, or jaws? (*N*, %)**					
Never	83 (92.2)	27 (90.0)	28 (93.3)	28 (93.3)	
Almost never	2 (2.2)	2 (6.7)	0 (0.0)	0 (0.0)	
Occasionally	4 (4.4)	0 (0.0)	2 (6.7)	2 (6.7)	0.236
Often	1 (1.1)	1 (3.3)	0 (0.0)	0 (0.0)	

Very often	0 (0.0)	0 (0.0)	0 (0.0)	0 (0.0)	
**(2) Have you experienced, or do you experience, an unpleasant taste due to problems with your teeth, mouth, or jaw? (*N*, %)**					
Never	66 (73.3)	24 (80.0)	19 (63.3)	23 (76.7)	
Almost never	14 (15.6)	4 (13.3)	5 (16.7)	5 (16.7)	
Occasionally	6 (6.7)	0 (0.0)	4 (13.3)	2 (6.7)	0.361
Often	1 (1.1)	0 (0.0)	1 (3.3)	0 (0.0)	
Very often	3 (3.3)	2 (6.7)	1 (3.3)	0 (0.0)	
**(3) Have you had, or do you have, severe pain in your mouth? (*N*, %)**					
Never	72 (80.0)	23 (76.7)	22 (73.3)	27 (90.0)	
Almost never	11 (12.2)	4 (13.3)	6 (20.0)	1 (3.3)	
Occasionally	2 (2.2)	2 (6.7)	0 (0.0)	0 (0.0)	0.199
Often	1 (3.3)	1 (3.3)	1 (3.3)	0 (0.0)	
Very often	3 (3.3)	0 (0.0)	1 (3.3)	2 (6.7)	
**(4) Have you been, or are you, uncomfortable eating certain types of food because of problems with your teeth, mouth, or jaws? (*N*, %)**					
Never	59 (65.6)	17 (56.7)	22 (73.3)	20 (66.7)	
Almost never	14 (15.6)	5 (16.7)	3 (10.0)	6 (20.0)	
Occasionally	10 (11.1)	4 (13.3)	3 (10.0)	3 (10.0)	0.825
Often	3 (3.3)	2 (6.7)	1 (3.3)	0 (0.0)	
Very often	4 (4.4)	2 (6.7)	1 (3.3)	1 (3.3)	

**(5) Were you aware that your teeth, mouth, and jaw "exist" (have you ever thought about them)? (*N*, %)**					
Never	43 (47.8)	16 (53.3)	14 (46.7)	13 (43.3)	
Almost never	5 (5.6)	2 (6.7)	2 (6.7)	1 (3.3)	
Occasionally	17 (18.9)	7 (23.3)	4 (13.3)	6 (20.0)	0.752
Often	5 (5.6)	0 (0.0)	3 (10.0)	2 (6.7)	
Very often	20 (22.2)	5 (16.7)	7 (23.3)	8 (26.7)	
**(6) Do you feel anxious about problems with your teeth, mouth, or jaws? (*N*, %)**					
Never	63 (70.0)	21 (70.0)	19 (63.3)	23 (76.7)	
Almost never	11 (12.2)	7 (23.3)	2 (6.7)	2 (6.7)	
Occasionally	8 (8.9)	1 (3.3)	5 (16.7)	2 (6.7)	0.162
Often	5 (5.6)	1 (3.3)	3 (10.0)	1 (3.3)	
Very often	3 (3.3)	0 (0.0)	1 (3.3)	2 (6.7)	
**(7) Do you think your diet is unsatisfactory due to problems with your teeth, mouth, or jaws? (*N*, %)**					
Never	75 (83.3)	25 (83.3)	26 (86.7)	24 (80.0)	
Almost never	6 (6.7)	3 (10.0)	0 (0.0)	3 (10.0)	
Occasionally	4 (4.4)	1 (3.3)	2 (6.7)	1 (3.3)	0.801
Often	2 (2.2)	0 (0.0)	1 (3.3)	1 (3.3)	
Very often	3 (3.3)	1 (3.3)	1 (3.3)	1 (3.3)	
**(8) Have you ever had to interrupt a meal due to problems with your teeth, mouth, or jaws? (*N*, %)**					

Never	75 (83.3)	26 (86.7)	25 (83.3)	24 (80.0)	
Almost never	4 (4.4)	1 (3.3)	1 (3.3)	2 (6.7)	
Occasionally	9 (10.0)	2 (6.7)	4 (13.3)	3 (10.0)	0.896
Often	0 (0.0)	0 (0.0)	0 (0.0)	0 (0.0)	
Very often	2 (2.2)	1 (3.3)	0 (0.0)	1 (3.3)	
**(9) Is it more difficult for you to relax due to problems with your teeth, mouth, or jaws? (*N*, %)**					
Never	73 (81.1)	24 (80.0)	24 (80.0)	25 (83.3)	
Almost never	7 (7.8)	5 (16.7)	0 (0.0)	2 (6.7)	
Occasionally	4 (4.4)	0 (0.0)	3 (10.0)	1 (3.3)	0.235
Often	4 (4.4)	1 (3.3)	2 (6.7)	1 (3.3)	
Very often	2 (2.2)	0 (0.0)	1 (3.3)	1 (3.3)	
**(10) Have you ever felt uncomfortable due to problems with your teeth, mouth, or jaws? (*N*, %)**					
Never	65 (72.2)	23 (76.7)	20 (66.7)	22 (73.3)	
Almost never	8 (8.9)	2 (6.7)	2 (6.7)	4 (13.4)	
Occasionally	9 (10.0)	3 (10.0)	4 (13.4)	2 (6.7)	0.660
Often	4 (4.4)	2 (6.7)	2 (6.7)	0 (0.0)	
Very often	4 (4.4)	2 (6.7)	2 (6.7)	2 (6.7)	
**(11) Were you irritable towards others due to problems with your teeth, mouth, or jaws? (*N*, %)**					
Never	81 (90.0)	26 (86.7)	27 (90.0)	28 (93.3)	
Almost never	3 (3.3)	1 (3.3)	1 (3.3)	1 (3.3)	

Occasionally	4 (4.4)	2 (6.7)	1 (3.3)	1 (3.3)	0.802
Often	1 (1.1)	1 (3.3)	0 (0.0)	0 (0.0)	
Very often	1 (1.1)	0 (0.0)	1 (3.3)	0 (0.0)	
**(12) Have you experienced difficulties performing daily tasks due to problems with your teeth, mouth, or jaws? (*N*, %)**					
Never	83 (92.2)	28 (93.3)	28 (93.3)	27 (90.0)	
Almost never	4 (4.4)	1 (3.3)	1 (3.3)	6 (6.7)	
Occasionally	2 (2.2)	1 (3.3)	1 (3.3)	0 (0.0)	0.741
Often	1 (1.1)	0 (0.0)	0 (0.0)	1 (3.3)	
Very often	0 (0.0)	0 (0.0)	0 (0.0)	0 (0.0)	
**(13) Do you feel that problems with your teeth, mouth, or jaws reduce your satisfaction with life? (*N*, %)**					
Never	75 (83.3)	25 (83.3)	26 (86.7)	24 (80.0)	
Almost never	2 (2.2)	0 (0.0)	0 (0.0)	2 (6.7)	
Occasionally	6 (6.7)	3 (10.0)	2 (6.7)	1 (3.3)	0.227
Often	3 (3.3)	2 (6.7)	1 (3.3)	0 (0.0)	
Very often	4 (4.4)	0 (0.0)	1 (3.3)	3 (10.0)	
**(14) Have you ever been unable to function at all due to problems with your teeth, mouth, or jaw? (*N*, %)**					
Never	82 (91.1)	26 (86.7)	29 (96.7)	27 (90.0)	
Almost never	5 (5.6)	3 (10.0)	0 (0.0)	2 (6.7)	
Occasionally	2 (2.2)	1 (3.3)	1 (3.3)	0 (0.0)	0.427
Often	0 (0.0)	0 (0.0)	0 (0.0)	0 (0.0)	

Very often	1 (1.1)	0 (0.0)	0 (0.0)	1 (3.3)

Data are presented as numbers and percentages. Group comparisons were performed using the chi-square test. Statistical significance was set at $p < 0.05$. Abbreviations: OHIP-CRO14, Croatian version of the Oral Health Impact Profile-14 questionnaire; HTP, heated tobacco product; N, number.
